# Defining Standard Data Reporting in Pelvic Exenterations for Non-Rectal Cancers: A Systematic Review of Current Data Reporting

**DOI:** 10.3390/cancers17183049

**Published:** 2025-09-18

**Authors:** PelvEx Collaborative

**Keywords:** pelvic exenteration, non-rectal cancer, outcomes, core outcome sets, locally advanced pelvic malignancies, locally recurrent pelvic cancer, locally advanced gynecological cancer, locally recurrent gynecological cancer, locally advanced urological cancer, locally recurrent urological malignancies, ovarian cancer, cervical cancer, vulvar cancer, vaginal cancer, prostate cancer, bladder cancer

## Abstract

**Simple Summary:**

Pelvic exenteration is a radical surgery for advanced and recurrent pelvic malignancies. Once a palliative procedure, it now offers a potential “cure” for a select cohort of patients. Advancement in the multi-disciplinary care of advanced pelvic malignancies has transformed oncological outcomes in these patients. This has transformed the field of pelvic exenteration surgery as there is a shift towards surgically removing these advanced malignancies previously deemed non-operable. This renewed interest has translated into an increase in the volume of literature reporting on exenteration surgery and heterogeneity in terms of outcome reporting. This review aims to systemically catalogue currently reported outcomes in the literature to highlight heterogeneity in outcome reporting and guide planned development of a standardized core information set following COMET guidelines for future reporting in the field.

**Abstract:**

Introduction: Pelvic exenteration (PEx) was first described in the 1940s as a palliative procedure in managing cervical cancer. Since then, advancements in perioperative care have transformed the options available to patients. This highly morbid procedure now offers a “cure” in a select cohort of patients with locally advanced and recurrent pelvic cancers. The large volume of literature in this field has resulted in a heterogeneity of data reporting, making comparative analysis extremely difficult. As such, we set out to examine the current literature and identify currently reported outcomes to guide development of a core information set (CIS) for data reporting for PEx in non-rectal cancers. Methods: A systematic review was carried out. Studies reporting on outcomes following PEx for advanced and recurrent gynecological, urological, and other non-rectal malignancies were included. Standardized outcomes were extracted and mapped to pre-determined domains. Results: Forty-four studies were found to meet our inclusion criteria. A total of 1735 data elements (DEs) were extracted verbatim, and these were assimilated into 111 standard DEs across nine domains. A wide range of reporting frequencies was observed, with the pathological domain containing the highest overall frequencies of DE reporting. Conversely, patient-reported and functional outcomes were noted to be the domain with the lowest frequency. Conclusions: This review highlights recent trends of increased reporting in the field of PEx and how this had invariably resulted in heterogeneous data reporting. We aim to guide the development of a CIS for reporting in non-rectal pelvic malignancies to help standardize future reporting.

## 1. Introduction

Pelvic exenteration (PEx) was first described by Brunschwig in the 1940s as a palliative procedure for the management of recurrent cervical cancer [1]. This was confined to a very selective cohort of patients for symptomatic palliation [2]. However, in the early series, PEx was associated with significant morbidity and a mortality rate of almost 25% [1].

Over the last few decades, with advances in anesthetic and peri-operative care, coupled with effective modern neo-adjuvant treatment strategies, outcomes following PEx have steadily improved. In select cases, PEx now offers a potential cure for advanced pelvic malignancies rather than symptom relief [3,4].

PEx procedures continue to be increasingly employed in the management of various pelvic malignancies [5], with R0 resection being the most important prognostic feature [2]. The wide variety of pelvic malignancies that are now eligible to undergo curative intent PEx has inevitably resulted in an increase in the volume of literature reporting on outcomes following PEx.

Key outcome reporting is an essential part of any research. The Core Outcomes Measured in Effectiveness Trial (COMET) Initiative aims to facilitate the formation and distribution of such key data elements in various areas of research. In the setting of complex surgery such as PEx where significant morbidity, survival differences, and patient quality of life are all high-stake consideration, essential and standardized core information sets (CIS) are key to ensure clinicians can recognize and counsel suitable patients pre-operatively, recognize patients who deviate early from a normal clinical course, and ensure that results from individual centers can be measured against international standards [6]. Furthermore, reporting using a CIS facilitates meta-analysis of data between various studies and centers to guide a better understanding in the field.

Currently, no CIS exists for reporting on PEx in either rectal or non-rectal advanced/recurrent pelvic malignancies. The aim of this study was to systematically review existing literature, demonstrate the large heterogeneity in individual outcome reporting, and formulate a standard set of outcomes in the field of advanced and recurrent non-rectal pelvic malignancies to help guide the future development of a CIS specific to these malignancies.

## 2. Methods

Our methodology was guided by the methods outlined in the COMET handbook as well as our previous study reporting on outcomes for PEx in rectal cancers. Our protocol detailing the steps involved in this study and future work to develop a CIS has been prospectively registered with the COMET initiative: https://www.cometinitiative.org/Studies/Details/3212 (Accessed on 1 September 2025).

### 2.1. Search Strategy

An electronic search of PubMed/Medline, Embase, Scopus, and the Cochrane Register of controlled trials was carried out. A search strategy in consultation with our subject librarian was formulated and included the following terms in combination with the Boolean operators AND/OR: “gynaecological” OR “cervical” OR “endometrial” OR “ovarian” OR “vulvar” OR “vaginal” OR “urological” OR “bladder” OR “prostate” AND “pelvic exenterations” OR “exenterations” OR “pelvectomy” OR “multi visceral resection” OR “multi organ resection”. The search was limited to papers published in English and published after the year 2000. The final search was completed on 25 August 2024.

### 2.2. Inclusion Criteria

Studies were included if they reported on surgical outcomes of PEx in non-rectal cancers and included more than 15 patients. Acceptable study designs included retrospective cohort studies, prospective cohort studies, randomized trials, cross-sectional studies, and qualitative studies.

### 2.3. Exclusion Criteria

Studies were excluded if rectal cancers accounted for >10% of the study population. Manuscripts where palliative exenterations accounted for >5% of the study population were also excluded.

Studies where the primary objective was non-surgical related outcome reporting (e.g., primarily focused on survival, operative technique) as well as studies where patients underwent cytoreductive surgery or any other concomitant surgical procedures besides PEx +/− simple metastectomy were excluded. Study designs excluded from our review included literature reviews, narrative reviews, conference abstracts, case reports, cohort studies, and studies reporting on fifteen patients or fewer.

### 2.4. Data Extraction

Extracted studies were imported to the COVIDENCE software (https://www.covidence.org/). All titles and abstracts were screened by two reviewers initially (MMS and CMD) before proceeding to full-text review of relevant papers. Any disagreements regarding inclusion or exclusion were resolved by a third reviewer (MEK). The same two reviewers proceeded to independently screen full-text articles to determine eligibility as guided by the above criteria. At each stage of the screening process, all reviews were performed independently, with disagreements (as highlighted by COVIDENCE) settled at the end of the review by a third senior reviewer until consensus was reached. Data element (DE) extraction from selected papers was carried out by three reviewers (MMS, CMD, and ND) who collected agreed-upon standardized DEs into an electronic extraction form developed using Microsoft Excel (Microsoft, Redmond, WA, USA).

### 2.5. Data Cataloging

Standardized DEs were created through several consensus meetings throughout the study period, where the three authors involved in the data extraction (MMS, CMD, and ND) agreed upon a term to capture DEs. These were extracted and cataloged as outlined previously on a data collection form developed on Microsoft Excel (Microsoft, Redmond, WA, USA). All standardized DEs were mapped to one of nine domains developed from those originally proposed by the COMET initiative but modified to suit our study.

### 2.6. Bias Assessment

Bias assessment on included studies was carried out using the ROBINS-I tool [7]. Studies were graded as being low risk (green), high risk (red), or unclear risk (yellow).

## 3. Results

**Figure 1:**
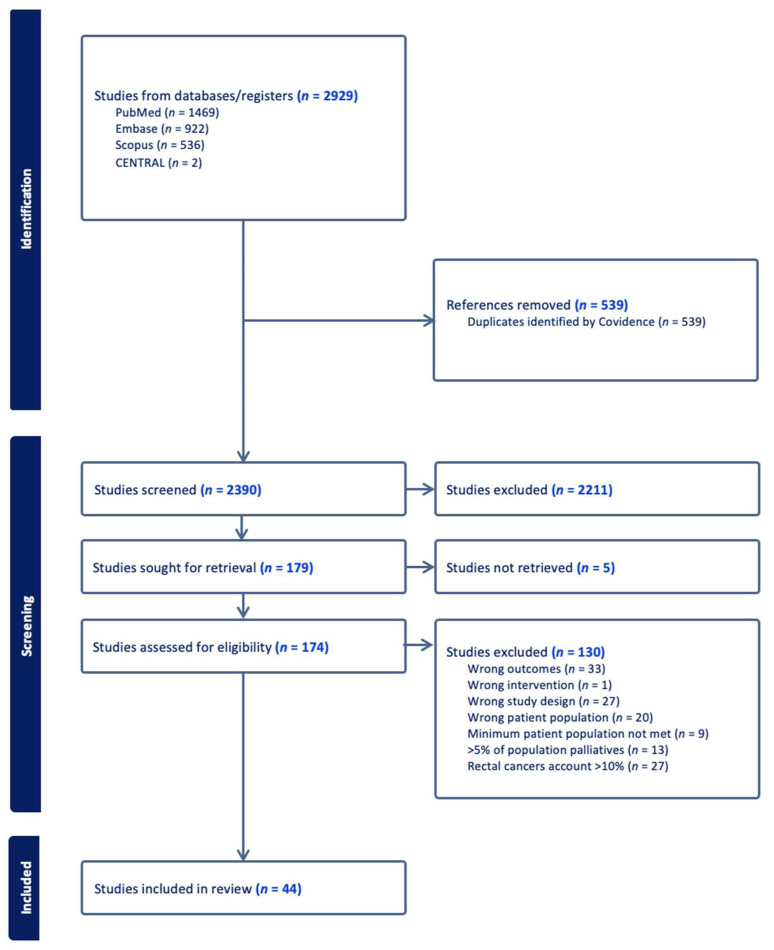
Flow diagram (using a PRISMA template) outlining progression of studies throughout the review process.

A total of 2929 studies were retrieved from our electronic search. There were 539 duplicates identified and removed. A total of 2390 studies were eligible for screening. After title and abstract screening, 174 studies were sought for full-text review. A total of 44 studies met our inclusion criteria [8,9,10,11,12,13,14,15,16,17,18,19,20,21,22,23,24,25,26,27,28,29,30,31,32,33,34,35,36,37,38,39,40,41,42,43,44,45,46,47,48,49,50,51] (Figure 1).

Included studies showed a trend of increased reporting since the turn of the last decade, with 77% of included studies published after 2011 (Table 1). Analyzing the geographical distribution of centers publishing in this field, we see that our included studies show a preponderance for centers in the USA, as these account for 34.1% of all included studies. A total of 12,786 patients were reported on across all included literature (range: 19–2647).

### 3.1. Data Element Reporting

A total of 1735 DEs were extracted verbatim from all included papers. These were mapped to 111 standard outcomes (Table 2), each of which was mapped to one of nine domains. Our core domains included “Patient Characteristics and Demographics”, “Pre-operative Assessment and Anesthetic Outcomes”, “Non-operative Treatment”, “Intra-operative/Surgical Outcomes”, “Pathological Outcomes”, “Reconstructive Outcomes”, “Post-operative Outcomes”, “Patient and Functional Outcomes”, and “Survival Outcomes”.

### 3.2. Study Bias

**Figure 2:**
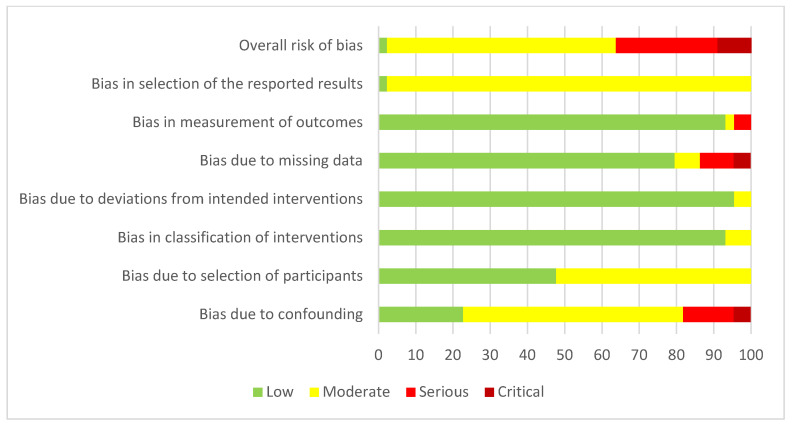
Risk of bias associated with observational studies (*n* = 44).

The risk of bias as assessed by the Robins-I tool is outlined in Figure 2. Sixteen studies were found to be of serious or critical bias [9,11,14,15,17,18,19,20,21,26,28,32,36,39,46,49].

The majority of bias was related to confounding factors (eight studies), followed by bias due to missing data (six studies) and bias in the measurement of outcomes (one study).

### 3.3. Patient Characteristics/Demographics

Standardized DEs mapped to this domain were overall the most reported DEs. Nine DEs fell under this heading, with tumor origin and patient median age being the DEs reported under this heading found in 44 (100%) and 42 (93.2%) studies, respectively. Other outcomes within this domain included patient body mass index (BMI) in 23 studies (52.3%), ethnicity in 9 studies (20.5%), gender in 24 studies (54.5%), presenting symptoms in 5 studies (11.4%), previous pelvic surgery in 7 studies (15.9%), and recurrent vs. primary tumor in 38 studies (86.4%).

### 3.4. Pre-Operative Assessment and Anesthetic Parameters

This domain was further divided into two subdomains, with three DEs mapped to serum markers and five outcomes mapped to the anesthetic subdomains.

Serum markers including albumin, hemoglobin, and creatinine levels pre-operatively were reported in two studies each (4.5%).

The anesthetic subdomain included ASA status as the most reported DE in seven studies (15.9%). Other DEs included the Charlson Co-morbidity Index in five studies (11.4%), smoking status in five studies (11.4%), ECOG status in one study (2.3%), and listings of individual co-morbidities in four studies (9.1%).

### 3.5. Non-Operative Treatments

As a domain, this was divided into three sub-domains, including “Neo-adjuvant treatments”, “Adjuvant Treatments”, and “Treatment Regimens”.

Neo-adjuvant treatments included neo-adjuvant chemotherapy in 15 studies (34.1%), neo-adjuvant radiotherapy in 25 studies (56.8%), neo-adjuvant chemo-radiotherapy in 12 studies (27.3%), and hormone therapy in 2 studies (4.5%).

Adjuvant treatment had three similar DEs to those seen in neo-adjuvant treatments with adjuvant chemotherapy reported in 16 studies (36.4%), adjuvant radiotherapy in 13 studies (29.5%), and adjuvant chemo-radiotherapy in 14 studies (31.8%).

Two DEs were mapped to the treatment regimen subdomains and included radiotherapy regimen/dose in 10 (22.7%) and chemotherapy in 6 (13.6%) studies, respectively.

### 3.6. Intra-Operative/Surgical Outcomes

The most common DE reported within this domain was exenteration type, reported in all 44 studies. Other standardized DEs within this domain included minimally invasive surgery: open vs. laparoscopic (vs robotic) in five studies (11.4%), bone resection in three studies (6.8%), major nerve resection in four studies (9.1%), and major vessel and muscle resection in one study (2.3%). Operative time was noted in 31 studies (70.5%), blood loss in 29 studies (65.9%), and volume of intra-operative transfusion in 22 studies (50%). Intra-operative mortality was noted in 5 studies (11.4%), and 14 papers recorded intra-operative complications (31.8%).

### 3.7. Pathological Outcomes

This domain included eleven DEs. Resection margins were the most commonly reported DE within this domain, with R0 and R1 resection reported in 29 and 27 studies, respectively. Other DEs included R2 resection in 19 studies (43.2%), tumor size in 18 studies (40.9%), tumor grade in eight studies (18.2%), and tumor stage in 15 studies (34.1%). Histological subtypes were reported in 25 studies (56.8%), nodal status in 20 papers (45.5%), lymphovascular invasion in six papers (13.6%), and perineural invasion in three papers (6.8%).

### 3.8. Reconstructive Outcomes

Ten DEs were associated with this domain. Reconstructive outcomes included use of flap reconstruction and flap reconstruction technique reported in 21 (47.7%) and 18 (40.9%) of studies, respectively. Use of bladder reconstruction reporting and bladder reconstruction techniques were both reported in 25 papers (56.8%).

Complications specific to reconstructive strategies were grouped under a subdomain and included flap complications in 7 studies (15.9%), urinary conduit complications in 16 studies (36.4), bowel anastomotic leaks in 12 studies (27.3%), and bowel reconstruction complications (not related to anastomotic leaks) in 6 studies (27.3%).

### 3.9. Post-Operative Outcomes

Two sub-domains were captured under this heading, including “Complications” and “Other post-operative Outcomes”.

Complications related to DEs were the outcomes with the most variation in reporting. Small bowel obstruction (SBO)/Ileus and need for re-operation were the most frequently reported complications in 33 (75%) and 31 (70.5%) studies, respectively. Twenty-five other complications were also mapped to this subdomain and included major complication (as defined by the Clavien–Dindo classification), minor complications (as defined by the Clavien–Dindo classification), infection, pneumonia, sepsis, wound complications, urinary tract infection, abscess (abdominal or pelvic), fistula, bowel perforation, bleeding/hematoma, thrombosed vascular graft, ulcers, renal complications, neurological complications, cardiovascular complications, respiratory complications (non-infectious), stoma complications, deep venous thrombosis (DVT), pulmonary embolism (PE), urethral obstruction, hernia, chyle leak, shock, and/or psychiatric complications. Corresponding frequency of reporting with regard to each of these can be found in Table 3.

Other post-operative DEs included length of hospital stay in 32 papers (72.7%), ICU stay/admission in 11 papers (25%), post-operative mortality (within 90 days) reported in 31 studies (70.5%), and re-admission in 6 studies (13.6%).

### 3.10. Patient-Reported and Functional Outcomes

This was the least reported on domain. Most DEs from this domain were mapped from two individual studies [37,41]. DEs captured included quality of Life (QoL) using QoL instruments, physical wellbeing, sexual wellbeing, social/role functioning, cognitive wellbeing, emotional wellbeing, GI symptom burden, respiratory symptom burden, neo-vagina satisfaction, and body image, all of which were reported in two studies (4.5%). Unspecified patient dissatisfaction was reported in three papers (6.8%).

### 3.11. Survival Outcomes

Ten DEs were mapped to the survival outcomes domain. These included median (or mean) follow-up in 23 papers (52.3%), median survival in 12 studies (27.3%), and overall survival in 32 studies (72.7%). Outcomes relating to disease recurrence under this domain included overall recurrence in 18 papers (43.2%), local recurrence in 8 studies (18.2%), distant recurrence in 7 studies (15%), and time to recurrence in 9 studies (20.5%). Other DEs included disease-free survival in 12 studies (29.5%), disease-specific survival in 2 studies (4.5%), and progression-free survival in 7 studies (15.9%).

## 4. Discussion

The complexity and associated morbidity of PEx necessitate a structured approach to patient assessment, selection, and follow-up. Standardization of data reporting is needed to ensure that real-world data is reproducible across centers and to facilitate benchmarking.

PEx has only emerged recently as the “standard” of care in managing patients with advanced and recurrent pelvic malignancies [52]. This may explain why there has been a recent increase in the volume of papers reporting on this field. Our review highlights the resultant increase in data reporting in the field of PEx for non-rectal pelvic malignancies, with a 467% increase in studies reporting on this field (three studies from 2000–2005 vs. 14 studies from 2020–2024). This has invariably resulted in increased heterogeneity in current data reporting. This has been a long-standing issue in the field of PEx, as demonstrated by Brown et al. previously [53].

The most consistently reported data relate to demographical factors, including patient age, tumor type, and primary vs. recurrence pattern of disease (93.2%, 100%, 86.4%). Other commonly reported data included compartments removed (100%), operative time (70.5%), and some pathological features, including R0 and R1 resection status (65.9% and 61.4%, respectively). These measures are all mostly oncological factors that would be expected to be reported on in any study examining outcomes following cancer resection procedures. However, we note that there exists variation in defining some of these measures among authors. For example, when describing R0 resection, some authors defined this as “microscopically clear”, while others were found to use variable margins ranging from 1 mm to 0.5 mm of clear tissue. We also note that there was variation in the terminology used. For example, “R0” was not explicitly mentioned in a considerable portion of studies, but rather inferred from the descriptions in the result section describing clear margins and non-clear margins. Other key outcomes that have a considerable impact on oncological outcomes, such as neo-adjuvant and adjuvant therapy, were overall poorly reported (i.e., neoadjuvant radiotherapy reported in 56.8% of studies and adjuvant chemotherapy reported in 36.4% of studies). Similarly, key outcomes relating to patients and their overall experience are poorly reported across the board. Patient quality of life was only assessed in two studies (4.5%). Given the current “curative intent” exenteration planned in the majority of cases, patient quality of life and impact on their day-to-day life are central when counselling and selecting patients. A standardized data set pertaining to the perioperative surgical care journey of PEx is therefore required to capture all these necessary details and to facilitate multi-institutional collaboration and outcome analysis.

With continued improvement in oncological care and outcomes, an increasing volume of patients with advanced and recurrent pelvic disease will be eligible for PEx procedures moving forward. This will undoubtedly add to the heterogeneity already present in the field. Meaningful synthesis of data across various international standards is necessary to define benchmarks for this complex procedure moving forward. Some authors have proposed benchmarks for PEx in rectal cancers [54] but none currently exist for PEx in the setting of non-rectal pelvic malignancies. Given the low volume of centers internationally performing these complex procedures, standardizing outcome definitions and developing a CIS is paramount to allow for multi-center data pooling, cross-analysis, and facilitate meta-analysis to define global benchmarks.

We do acknowledge some limitations to our study, including our inclusion of a diverse set of pelvic malignancies under the single heading of “non-rectal pelvic” malignancies. We also acknowledge our strict inclusion criteria, which selected for larger studies and also excluded studies that included a large number of rectal cancers or palliative cases. These exclusions may have led to the omission of additional potential outcome variables. Nonetheless, we were able to include a considerable number of studies and include a large volume of outcomes that were standardized to relevant domains, capturing the perioperative care journey of patients with non-rectal pelvic malignancies undergoing PEx.

## 5. Conclusions

Our study demonstrated the increased volume of literature in the field of PEx in non-rectal malignancies as well as the wide spectrum of outcomes currently reported. We demonstrate that standardization of data is possible, but also acknowledge that current data reporting may not be representative of those essential in defining global benchmarks. A standardized data set should include long- and short-term outcomes that are pertinent to clinicians but also capture the patient experience to reflect the complex nature of these procedures. We hope that by standardizing the current data being reported in the literature, this will guide us in carrying out a Delphi study in order to develop a CIS for reporting on PEx in advanced and recurrent non-rectal pelvic cancers.

## Figures and Tables

**Table 1:** Study Characteristics.

**Publication Period**	
2000–2005	3
2006–2010	7
2011–2015	9
2016–2020	11
2021–2024	14
**Geographical Distribution**	
North America	16
South America	1
Europe	20
Asia	6
International multi-center	1

**Table 2:** Domains and Mapped Data Elements (*n* = 111).

Domain	Number of Outcomes
Patient Characteristics and Demographics	9
Pre-operative Assessment and Anesthetic Parameters	8
Non-operative Treatment	9
Intra-operative/Surgical	12
Pathological Outcomes	11
Reconstructive Outcomes	10
Post-Operative Outcomes	31
Patient Reported and Functional Outcomes	11
Survival Outcomes	10

**Table 3:** PEx Standardized Domains and Data Elements.

Domain	Outcome	Number (%)
Patient Characteristics and Demographics	Age	41 (93.2)
	Ethnicity	9 (20.5)
	Gender	24 (54.5)
	Socioeconomic status	5 (11.4)
	BMI	23 (52.3)
	Tumor origin	44 (100)
	Recurrent vs. Primary tumor	38 (86.4)
	Presenting symptom	5 (11.4)
	Previous pelvic surgery	7 (15.9)
Pre-operative Assessment and Anesthetic Parameters	**Serum Markers**	
	Albumin level pre-op	2 (4.5)
	Hemoglobin level pre-op	2 (4.5)
	Creatinine level pre-op	2 (4.5)
	**Pre-operative Anesthetic**	
	ASA status	7 (15.9)
	ECOG	1 (2.3)
	Charlson Comorbidity Index	5 (11.4)
	Smoking status	5 (11.4)
	Individual co-morbidities listed	4 (9.1)
Non-operative Treatment	**Neo-adjuvant Treatment**	
	Neo-adjuvant Chemotherapy	15 (34.1)
	Neo-adjuvant Radiotherapy	25 (56.8)
	Neo-adjuvant chemotherapy + radiotherapy	12 (27.3)
	**Adjuvant Treatment**	
	Adjuvant Chemotherapy	16 (36.4)
	Adjuvant Radiotherapy	13 (29.5)
	Adjuvant Chemotherapy + Radiotherapy	14 (31.8)
	Hormone Therapy	2 (4.5)
	**Treatment Regimens**	
	Radiotherapy regimen/dose	10 (22.7)
	Chemotherapy Regimen	6 (13.6)
Intra-operative/Surgical	**Surgical Outcomes**	
	Compartments/Exenteration Type	44 (100)
	MIS; Open vs. Laparoscopic (vs robotic)	5 (11.4)
	Bone resection (pelvis/sacrum)	3 (6.8)
	Major nerve resection	4 (9.1)
	Major vessel resection	1 (2.3)
	Major muscle resection	1 (2.3)
	operative time	31 (70.5)
	Blood loss	29 (65.9)
	Volume of transfusion intra-op	22 (50)
	Intra-operative Mortality	5 (11.4)
	Intra-operative Complications	14 (31.8)
	**Adjuncts to Operation**	
	IORT	8 (18.2)
Pathological Outcomes	R0 resection	29 (65.9)
	R1 resection	27 (61.4)
	R2 resection	19 (43.2)
	Tumor size	18 (40.9)
	Tumor Grade	8 (18.2)
	Tumor Stage	15 (34.1)
	FIGO Stage	11 (25)
	Histological Subtype	25 (56.8)
	Nodal status	20 (45.5)
	Lymphovascular invasion	6 (13.6)
	Perineural invasion	3 (6.8)
Reconstructive Outcomes	Flap reconstruction	21 (47.7)
	Flap reconstruction technique	18 (40.9)
	Bladder reconstruction	25 (56.8)
	Bladder reconstruction technique	25 (56.8)
	Bowel reconstruction	21 (47.7)
	Vaginal reconstruction	19 (43.2)
	**Reconstructive complications**	
	Flap complications	7 (15.9)
	Urinary conduit complications	16 (36.4)
	Anastomotic leak	12 (27.3)
	Bowel reconstruction complications (non-leak)	6 (13.6)
Post-Operative Outcomes	**Complications**	
	Major complications (as defined by paper)	23 (52.3)
	Minor complications (as defined by paper)	12 (27.3)
	Re-operation	31 (70.5)
	Infection	29 (65.9)
	Pneumonia	11 (25)
	Sepsis	24 (54.5)
	Wound complications	21 (47.7)
	Urinary tract infection	17 (38.6)
	Abscess (abdominal or pelvic)	26 (59.1)
	SBO/Ileus	33 (75)
	Fistula	30 (68.2)
	Bowel perforation	9 (20.5)
	Bleeding/Hematoma	10 (22.7)
	Thrombosed Vascular Grafts	1 (2.3)
	Ulcers	4 (9.1)
	Renal Complications	20 (45.5)
	Neurological complications	9 (20.5)
	Cardiovascular complications	15 (34.1)
	Respiratory complications (non-infectious)	7 (15.9)
	Stoma Complications	12 (27.3)
	DVT	20 (45.5)
	PE	20 (45.5)
	Urethral obstruction	9 (20.5)
	Hernia	1 (2.3)
	Chyle leak	1 (2.3)
	Shock	3 (6.8)
	Psychiatric	1 (2.3)
	**Other:**	
	Length of hospital stay	32 (72.7)
	ICU stay / ICU Admission	11 (25)
	Post-operative mortality (within 90 days)	31 (70.5)
	Re-admission (within 90 days)	6 (13.6)
Patient Reported and Functional Outcomes	QoL (Using QoL Instruments)	2 (4.5)
	Physical wellbeing	2 (4.5)
	Sexual wellbeing	2 (4.5)
	Social/Role Functioning	2 (4.5)
	Cognitive Functioning	2 (4.5)
	Emotional Wellbeing	2 (4.5)
	GI Symptom Burden	2 (4.5)
	Respiratory Symptom Burden	2 (4.5)
	Unspecified patient dissatisfaction	3 (6.8)
	Neo-vagina Satisfaction	2 (4.5)
	Body Image	2 (4.5)
Survival Outcomes	Median (OR mean) time of follow up	23 (52.3)
	Recurrence overall	19 (43.2)
	Local recurrence	8 (18.2)
	Distant recurrence	7 (15)
	Overall survival	32 (72.7)
	Median survival	12 (27.3)
	Disease-free survival	13 (29.5)
	Disease-specific survival	2 (4.5)
	Progression-free survival	7 (15.9)
	Time to recurrence	9 (20.5)

## Data Availability

The raw data supporting the conclusions of this article will be made available by the authors on request.

## Appendix A. Full Authorship

Salama MM, Ryan ÉJ, Doherty CM, Dunne N, Ryan OK, Aalbers AGJ, Abdul Aziz N, Abecasis N, Abern M, Abraham-Nordling M, Akiyoshi T, Alahmadi R, Alberda W, Albert M, Andric M, Angeles M, Angenete E, Antoniou A, Apte S, Armitage J, Auer R, Austin KK, Aytac E, Aziz O, Bacalbasa N, Baker RP, Bali M, Baransi S, Baseckas G, Bebington B, Bedford M, Bednarski BK, Beets GL, Belli F, Berg PL, Bergzoll C, Beynon J, Biondo S, Boyle K, Bordeianou L, Brecelj E, Bremers AB, Brown KGM, Brunner M, Buchwald P, Bui A, Burgess AN, Burger JWA, Burling D, Burns EM, Byrne CM, Campain N, Canda AE, Carvalhal S, Castro L, Caycedo-Marulanda A, Ceelen W, Chan KKL, Chang GJ, Charles R, Chew MH, Chok AK, Choi GS, Chong P, Christensen HK, Clark D, Clouston H, Collins D, Colquhoun AJ, Constantinides J, Corr A, Coscia M, Cosimelli M, Cotsoglou C, Coyne PE, Croner RS, Damjanovic L, Daniels IR, Davies M, Davies RJ, Delaney CP, Delisle M, Demirli S, de Wilt JHW, Denost QD, Deutsch C, Dietz D, Domingo S, Dozois EJ, Drozdov E, Duff M, Edmundson A, Egger EK, Eglinton T, Enrique-Navascues JM, Espín-Basany E, Evans MD, Eyjólfsdóttir B, Fahy M, Fearnhead NS, Ferenschild F, Fichtner-Feigl S, Fidlers T, Flatmark K, Fleming FJ, Flor-Lorente B, Folkesson J, Foskett K, Frizelle FA, Funder JA, Gallego MA, Garcia Aguilar J, García-Granero A, García-Granero E, García-Sabrido JL, Gargiulo M, Gava VG, Gentilini L, George ML, George V, Georgiou P, Ghosh A, Ghouti L, Gil-Moreno A, Giner F, Ginther N, Glover T, Glyn T, Goffredo P, Golda T, Gögenur I, Gonzalez-Argente FX, Griffiths B, Gronchi A, Grotenhuis BA, Guerra G, Gwenaël F, Harris C, Harris DA, Hagemans JAW, Hanchanale V, Harji DP, Helbren C, Helewa RM, Hellawell G, Heriot AG, Hochman D, Hohenberger W, Holm T, Holmström A, Hompes R, Hornung B, Hurton S, Hyun E, Ito M, Iversen LH, Jalbuu G, Jeri-McFarlane S, Jenkins JT, Johnstone CSH, Jourand K, Kaffenberger S, Kandaswamy GV, Kapur S, Kanemitsu Y, Kaufman M, Kazi M, Kaul S, Kelley SR, Keller DS, Kelly ME, Kersting S, Ketelaers SHJ, Khan MS, Khaw J, Kim H, Kim HJ, Kiran R, Koh CE, Kok NFM, Kokelaar R, Kontovounisios C, Kose F, Koutra M, Kraft M, Kristensen HØ, Kumar S, Kusters M, Lago V, Lakkis Z, Lampe B, Langheinrich MC, Larach TT, Larkin J, Larsen SG, Larson DW, Law WL, Laurberg S, Lee PJ, Limbert M, Loria A, Lydrup ML, Lyons A, Lynch AC, Lynch N, Mackintosh M, Maciel J, Malakorn S, Manfredelli S, Mann C, Mantyh C, Mathis KL, Margues CFS, Martinez A, Martling A, Meijerink WJHJ, Merchea A, Merkel S, Mehta AM, McArthur DR, McCormick JJ, McCormick P, McDermott FD, McGrath JS, McPhee A, Maciel J, Malde S, Mirnezami A, Manfredelli S, Martinez-Gomez C, Mikalauskas S, Modest DP, Monson JRT, Morton JR, Mullaney TG, Nair R, Navarro AS, Neeff H, Negoi I, Neto JWM, Nguyen B, Nielsen MB, Nieuwenhuijzen GAP, Nilsson PJ, Nordkamp S, O’Dwyer ST, Oates J, Paarnio K, Palmer G, Pappou E, Park J, Patsouras D, Paty PB, Peacock A, Pellino G, Peterson AC, Peulen HMU, Pfeffer F, Piqeur F, Pinson J, Poggioli G, Polignano F, Proud D, Quinn M, Oliver A, Quyn A, Radwan RW, Rajendran N, Rasheed S, Rasmussen PC, Rausa E, Raza I, Regenbogen SE, Reims HM, Renehan A, Rintala J, Rocha R, Rochester M, Rohila J, Rogers A, Rothbarth J, Rottoli M, Roxburgh C, Rutten HJT, Rydbeck D, Safar B, Sagar PM, Sahakitrungruang C, Sahai A, Saklani A, Sammour T, Sayyed R, Schizas AMP, Schwarzkopf E, Scripcariu D, Scripcariu V, Seifert G, Sekhar H, Selvasekar C, Shaban M, Shaikh I, Simianu VV, Simillis C, Simpson JAD, Skeie-Jensen T, Smart NJ, Smart P, Smith JJ, Smith TG, Sokmen S, Solbakken AM, Solomon MJ, Sørensen MM, Sorrentino L, Spasojevic M, Steele SR, Steffens D, Stitzenberg K, Stocchi L, Stylianides NA, Swartling T, Sumrien H, Sutton PA, Swartking T, Takala H, Tan EJ, Taylor C, Taylor D, Tejedor P, Tekin A, Tekkis PP, Teras J, Thanapal MR, Thaysen HV, Thorgersen EB, Thurairaja R, Tiernan J, Toh EL, Tsarkov P, Tolenaar JL, Tsukada Y, Tsukamoto S, Tuech JJ, Turner G, Turner WH, Tuynman JB, Valente M, van Ramshorst GH, Van Nieuwenhove Y, van Rees JM, Vásquez-Jiménez W, Vather R, Verhoef C, Vierimaa M, Vizzielli G, Voogt ELK, Uehara K, Wagstaff M, Wakeman C, Warrier S, Wasmuth HH, Waters P, Weber K, Weiser MR, West CT, West MA, Westney OL, Wheeler JMD, Wild J, Wilson M, Wolthuis A, Yano H, Yip B, Yip J, Yoo RN, Zappa MA, Winter DC, Kelly ME.
